# Wedelolactone facilitates Ser/Thr phosphorylation of NLRP3 dependent on PKA signalling to block inflammasome activation and pyroptosis

**DOI:** 10.1111/cpr.12868

**Published:** 2020-07-12

**Authors:** Hao Pan, Yuqing Lin, Jianping Dou, Zhen Fu, Yanqing Yao, Shanyu Ye, Saixia Zhang, Neng Wang, Aijun Liu, Xican Li, Fengxue Zhang, Dongfeng Chen

**Affiliations:** ^1^ Research Center for Integrative Medicine School of Basic Medical Sciences Guangzhou University of Chinese Medicine Guangzhou P.R China; ^2^ Dongguan & Guangzhou University of Chinese Medicine Cooperative Academy of Mathematical Engineering for Chinese Medicine Dongguan P.R China; ^3^ Department of Human Anatomy School of Basic Medical Sciences Guangzhou University of Chinese Medicine Guangzhou P.R China; ^4^ Center for Experimental Teaching School of Basic Medical Sciences Guangzhou University of Chinese Medicine Guangzhou P.R China; ^5^ School of Chinese Herbal Medicine Guangzhou University of Chinese Medicine Guangzhou P.R China

**Keywords:** gouty arthritis, NLRP3 phosphorylation, PKA, pyroptosis, Wedelolactone

## Abstract

**Objectives:**

Wedelolactone exhibits regulatory effects on some inflammatory diseases. However, the anti‐inflammatory mechanism of wedelolactone has not been entirely unravelled. Therefore, the present study focuses on investigating the mechanism of wedelolactone on NLRP3 inflammasome in macrophages and its influence on MSU‐induced inflammation.

**Materials and Methods:**

BMDM, J774A.1 and PMA‐differentiated THP‐1 macrophages were primed with LPS and then stimulated with ATP or nigericin or MSU crystal in the presence or absence of wedelolactone. The cell lysates and supernatants were collected to detect NLRP3 inflammasome components such as NLRP3, ASC and caspase 1, as well as pyroptosis and IL‐1β production. In addition, the anti‐inflammatory effects of wedelolactone on MSU‐induced peritonitis and arthritis mice were also evaluated.

**Results:**

We found that wedelolactone broadly inhibited NLRP3 inflammasome activation and pyroptosis and IL‐1β secretion. Wedelolactone also block ASC oligomerization and speck formation. The inhibitory effects of wedelolactone were abrogated by PKA inhibitor H89, which also attenuated wedelolactone‐enhanced Ser/Thr phosphorylation of NLRP3 at PKA‐specific sites. Importantly, wedelolactone could abate MSU‐induced IL‐1β production and neutrophils migration into peritoneal cavity, and reduced caspase 1 (p20) and IL‐1β expression in the joint tissue of MSU‐induced arthritis.

**Conclusion:**

Our results indicate that wedelolactone promotes the Ser/Thr phosphorylation of NLRP3 to inhibit inflammasome activation and pyroptosis partly through potentiating PKA signalling, thus identifying its potential use for treating MSU‐induced peritonitis and gouty arthritis.

## Graphical abstract

Wedelolactone is a major active ingredient of the traditional medicinal herb eclipta, which demonstrates a broad‐spectrum inhibition effect on NLRP3 inflammasome activation triggered by multiple stimuli. Wedelolactone suppresses NLRP3 inflammasome assembly, pyroptosis and IL‐1β secretion through heightening PKA‐dependent NLRP3 phosphorylation. Importantly, wedelolactone could alleviate NLRP3‐related inflammation in MSU‐induced peritonitis and gouty arthritis.

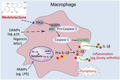

## 1 INTRODUCTION

Wedelolactone is a major active chemical ingredient derived from leaves of the Eclipta alba, which is applied as a traditional medicinal plant in America, Asia, and Africa.^1^ Recent studies have demonstrated that wedelolactone exhibits broad pharmacological effects, such as hepatoprotective,^2^ antioxidant,^3^, ^4^, ^5^ anti‐tumour^6^ and anti‐inflammatory such as enteritis.^7^, ^8^, ^9^ Wedelolactone has been declared to suppress NF‐κB‐mediated transcription of inflammatory genes, such as interleukin (IL)‐1β, IL‐6 and tumour necrosis factor (TNF)‐α.^10^ Although the anti‐inflammatory activity of wedelolactone is emerging, the underlying mechanisms are not clearly revealed.

NLRP3 (NLR family pyrin domain containing 3) inflammasome is one of the most characteristic inflammasome and plays an essential role in regulating immune and inflammatory responses. NLRP3 inflammasome is a protein complex composed of NLRP3, ASC (apoptosis‐associated speck‐like protein containing a caspase recruitment domain) and pro‐caspase‐1. Activation of NLRP3 inflammasome involves two processes. TLR agonist (such as LPS) firstly stimulates the NF‐κB pathway to promote NLRP3 and pro‐IL‐1β expression. Then, the pathogen‐associated molecular patterns (PAMP) or damage‐associated molecular patterns (DAMP), such as extracellular adenosine triphosphate (ATP), nigericin and monosodium urate (MSU) crystals (gout aetiology), can activate the NLRP3 inflammasome.^11^ The assembly of this complex induces the activation of caspase‐1, which promotes the cleavage of pro‐IL‐1β to generate mature IL‐1β. Concomitantly, the active caspase‐1, a (p20/p10) 2 tetramer, is necessary and sufficient for cleavage of gasdermin D (GSDMD) to generate N‐terminal fragment (GSDMD‐NT). GSDMD‐NT forms pore in the plasma membrane, which can regulate IL‐1β secretion and inflammatory form of cell death named pyroptosis.^12^, ^13^, ^14^ The inflammatory cytokines such as IL‐1β can recruit the neutrophils and macrophage to enhance immune response. Excessive activation of NLRP3 inflammasome contributes to the progress of several immune diseases, such as ulcerative colitis, endotoxic shock and gouty arthritis.^15^, ^16^

Multiple proteins and signalling pathways have been strongly linked to the modulation of NLRP3 inflammasome activation in macrophages.^17^, ^18^, ^19^ Gong, T *et al* have summarized that NLRP3 phosphorylation modification regulates inflammasome assembly and activation by targeting different inflammasome components.^20^ For instance, PKA specifically phosphorylates NLRP3 at Ser/Thr residue, which induces NLRP3 ubiquitination and the suppression of NLRP3 inflammasome.^21^, ^22^, ^23^ And yet JNK1‐mediated NLRP3_S194_ phosphorylation regulates NLRP3 deubiquitination and facilitates inflammasome assembly.^24^ The phosphorylation modification of ASC governs the formation of ASC speck. Syk and JNK control ASC phosphorylation and modulate the ASC‐dependent inflammasome activation.^25^ Our previous work has found that several natural compounds can inhibit NLRP3 inflammasome activation and pyroptosis to suppress the inflammatory response.^26^, ^27^ Therefore, it is feasible to modulate NLRP3 inflammasome activation and pyroptosis by targeting the phosphorylation of inflammasome components to treat inflammation‐related diseases.

Monosodium urate (MSU) crystal can stimulate the activation of NLRP3 inflammasome and the secretion of inflammatory cytokines such as IL‐1β, which can induce the progress of gout.^28^, ^29^, ^30^ The microtubule skeleton drug colchicine, as the therapeutic drugs for gout, mitigates the gout flare by inhibiting NLRP3 inflammasome activation.^31^ Other natural compounds such as quercetin, catechin and EGCG have also displayed the potential to ameliorate arthritis.^32^ Therefore, NLRP3 inflammasome inhibitors, a new type of anti‐inflammatory drug candidate that is different from the directly blocking IL‐1β, promise to alleviate arthritis symptoms and limit gout flare. However, the detailed mechanism of wedelolactone regulating NLRP3 inflammasome and pyroptosis has not been fully clarified, and the influence on gouty arthritis has not been addressed before.

In the present study, our results showed that wedelolactone suppressed NLRP3 inflammasome activation, pyroptosis and IL‐1β release in the macrophage. Such wedelolactone‐mediated inhibition of NLRP3 activation and ASC speck formation was abrogated by the PKA pathway inhibitor, indicating the involvement of PKA signalling in this process. To further support of this, the Ser/Thr phosphorylation of NLRP3 at PKA‐specific site was markedly increased by wedelolactone, but was blocked by PKA inhibitor H89. Moreover, wedelolactone could attenuate the MSU‐induced peritonitis and gouty arthritis, suggesting that it also prevented NLRP3 activation in vivo. Our results conclude that wedelolactone can suppress NLRP3 inflammasome activation and pyroptosis through heightening PKA‐induced NLRP3 phosphorylation to exhibit therapeutic effects against NLRP3‐related inflammatory diseases.

## 2 MATERIALS AND METHODS

### 2.1 Animals

C57BL/6J male mice (8‐10 weeks old) were bought from the laboratory animal centre of Guangzhou University of Chinese Medicine. After acclimatized for one week, mice were sustained to the light/dark cycle for 12 hours at 22 ~ 24°C and given free access to food and water. All animal experiment protocols were in strict guidelines with good animal practice as defined by the Animal Care Committee of Guangzhou University of Chinese Medicine.

### 2.2 Chemicals and antibodies

Wedelolactone (T3384), colchicine (T0320) and H89 (T6250) were acquired from TargetMol (Shanghai, China). Dulbecco's modified Eagle's medium (DMEM) medium with high glucose, RPMI 1640 medium, Opti‐MEM, foetal bovine serum (FBS), streptomycin/penicillin and MitoSOX (M36008) was obtained from ThermoFisher (California, USA). Lipopolysaccharide (LPS, L4391), adenosine triphosphate (ATP, A6419), propidium iodide (PI, P4170), Hoechst 33342 (B2261), dimethyl sulphoxide (DMSO, D8418) and phorbol 12‐myristate 13‐acetate (PMA, P8139) were bought from Sigma‐Aldrich (Missouri, USA). Monosodium urate (MSU) crystal (tlrl‐msu) and nigericin (tlrl‐nig) were from InvivoGen (California, USA). Cell lysis buffer for Western and IP (P0013), phenylmethanesulfonyl fluoride (PMSF) (ST506), BeyoECL Plus (P0018AM), Hematoxylin and Eosin (H&E) Staining Kit (C0105) and Lactate Dehydrogenase (LDH) Cytotoxicity Assay Kit (C0017) were bought from Beyotime Biotechnology (Haimen, China). BCA assay kit (FD2001) was from Fude Biotechnology (Hangzhou, China). Polyvinylidene difluoride (PVDF) membrane (0.22 μm, ISEQ00010) was from Merck Millipore (Darmstadt, Germany). Protein G agarose beads (#37478), Alexa Fluor 488 goat anti‐rabbit IgG (#4412) and Alexa Fluor 555 goat anti‐mouse IgG (#4409) antibodies, the antibody against NLRP3 (#15101), ASC (#67824), p‐(Ser/Thr) PKA substrate (#9621), Phospho‐SAPK/JNK (#4668) and Phospho‐AMPKα (#2535) were from Cell Signaling Technology (CST) (Massachusetts, USA). Anti‐GAPDH (ab181602), anti‐GSDMD (ab209845) and anti‐IL‐1β (ab9722) were purchased from Abcam (Cambridge, United Kingdom). Anti‐caspase‐1 (p20) antibody (AG‐20B‐0042) was from Adipogen AG (Liestal, Switzerland). Anti‐caspase‐1 (NB100‐56565) antibody was from Novus Biologicals (Colorado, USA). CD11b‐FITC (557 396), F4/80‐PE (565 410) and Ly6G‐Percp‐Cy5.5 (560 062) were from BD Biosciences (Franklin Lakes, NJ, USA). Mouse IL‐1β (EMC001b) and TNF‐α (EMC102a) ELISA kits were from Neobioscience Technology Company (Shenzhen, China). SP Kit (Broad Spectrum) (SP‐0022) and DAB Horseradish Peroxidase Color Development Kit (C‐0010) were obtained from Bioss (Beijing, China).

### 2.3 Cell culture and stimulation

J774A.1, L929 and THP‐1 cell lines were from the Chinese academy of sciences cell banks. J774A.1 and L929 cells were cultured in Dulbecco's modified Eagle's medium (DMEM) supplemented with 10% FBS, 100 U/ml penicillin and 100 μg/ml streptomycin. THP‐1 cells were cultured in RPMI 1640 medium supplemented with 10% FBS and antibiotics. THP‐1 cells were differentiated into macrophages by incubation with 100 nM PMA for 3 h.

To acquire bone marrow‐derived macrophages (BMDMs),^33^ the femur and tibia were collected from C57BL/6J mice, and bone marrow cells were flushed with complete DMEM containing 1% FBS and antibiotics. Erythrocytes were removed via treatment with red blood cell lysis buffer, and the cell suspensions were filtered through a 40‐μm cell strainer for the removal of any cell clumps. Single‐cell suspension was then cultured for 2 h at 37°C, and non‐adherent cells were collected and seeded in complete DMEM with 20% medium conditioned by L929 mouse fibroblasts. For full differentiation of BMDMs, the cells were cultured for an additional 7 days with replacement of the medium every 2 days. The BMDM purity was assessed by flow cytometry using CD11b and F4/80 antibodies and was routine > 95%.

To stimulate NLRP3 inflammasome activation, macrophages were plated in 24‐well or 6‐well plates. After cultured overnight, the medium was changed to Opti‐MEM with 1% FBS and cells were primed with 500 ng/ml LPS for 4 h, and wedelolactone was added for another 30 min. Cells were then stimulated with ATP and nigericin (10 μM) for 1 h or MSU (200 μg/ml) for 5 h. The supernatants and cell lysates were collected and analysed caspase‐1 activation and IL‐1β secretion by immunoblotting.

### 2.4 Flow cytometry assays

Flow cytometry analysis was implemented as previously described.^34^ BMDMs or peritoneal cells were stained for 30 min with the appropriate fluorescence‐conjugated antibodies. Then, cells were washed and resuspended with flow cytometry staining buffer (3% FBS in PBS). The cells were stained with CD11b‐FITC, F4/80‐PE and/or Ly6G‐Percp‐Cy5.5, and then, the cell phenotype was analysed on a flow cytometer (BD Biosciences). Flow cytometry data were plotted and quantified by FlowJo software (TreeStar).

### 2.5 Enzyme‐linked immunosorbent assay (ELISA)

The culture supernatant of cells or tissues was collected and assayed by Mouse ELISA kit according to the manufacturer's instructions. The inflammatory cytokine concentration was measured at 450 nm wavelength of absorbance and calculated by GraphPad Prism 8 linear regression analysis.

### 2.6 Western Blotting

Western blotting was performed as described previously.^35^ Medium supernatants from treated macrophages were precipitated. Cells were harvested by cell lysis buffer. Total protein concentration was determined by the BCA assay. Equal amounts of protein were loaded on SDS‐polyacrylamide gel, transferred onto PVDF membranes and blocked in 5% skimmed milk for 1 h. Subsequently, PVDF membranes were incubated in the presence of the primary antibody against NLRP3, Caspase‐1, ASC, IL‐1β, GSDMD, Caspase‐1 (p20) and GAPDH overnight at 4 ℃ and followed by HRP‐conjugated secondary antibody incubation for 1 h. The protein expression was visualized using an enhanced chemiluminescence kit and photographed by the Tanon 4600 automatic chemiluminescence image analysis system (Tanon Science and Technology Co., Ltd., Shanghai, China).

### 2.7 Evaluation of cell death

To examine inflammatory cell death (also known as pyroptosis), cells were plated in 24‐well plate overnight and stimulated with LPS for 4 h. After that, wedelolactone was added for another 30 min. Cells were stimulated by ATP or nigericin and stained with PI (2 µg/ml) plus Hoechst 33342 (5 µg/ml) for 10 min. Images of pyroptosis cells were immediately captured using IN Cell Analyzer 2000 with High Content Analysis (GE Healthcare). All data were representative of at least ten randomly selected image fields. PI‐positive cells revealed cell death.

LDH release assay was also applied to analyse cell death. Macrophages were seeded in plate overnight and stimulated as described above. The supernatants of the cells were collected. 120 μl of supernatant sample was transferred to a 96‐well plate. 60 μl of the LDH Cytotoxicity Assay Kit reagent was added to each well and incubated for 30 min. The absorbance signal was measured at 490 nm in a Microplate Reader. Cytotoxicity was calculated according to the manufacturer's instructions.

### 2.8 Immunofluorescence staining and imaging

Immunofluorescence analysis was conducted as previously described.^36^ In short, macrophages were adhered to coverslip in 24‐well plate overnight and stimulated as described above, and stained with MitoSOX (5 µM), then cells were washed three times with PBS and fixed with 4% paraformaldehyde for 15 min. After that, cells were washed with PBST three times. Genomic DNA was stained with Hoechst 33342. Fluorescence images were carried out by using a Zeiss LSM 800 confocal laser scanning microscope.

### 2.9 ASC oligomerization and ASC speck formation

LPS‐primed BMDMs were plated at a density of 2 × 10^6^/well in 6‐well plate and treated with inflammasome inducers in the presence of wedelolactone for the indicated time. The supernatants were removed, and then, cells were lysed by ice‐cold PBS containing 0.5% Triton‐X 100 for 30 min. Lysates were centrifuged at 6,000 × g for 15 min at 4°C. The pellets were washed twice in 1 ml ice‐cold PBS and resuspended in 200 µl PBS. 2 mM disuccinimidyl suberate (from a stock 100 mM DSS solution in DMSO) was added to the resuspended pellets, which were incubated at room temperature for 30 min with rotation. Samples were then centrifuged at 6000 × g for 15 min at 4°C. The cross‐linked pellets were resuspended in 30 µl 1 × SDS loading buffer and then boiled for 5 min and analysed by Western blotting with the anti‐ASC antibody.

To observe the formation of ASC speck, BMDMs were treated with the indicated reagents. Macrophages were fixed in 4% paraformaldehyde for 15 min and permeabilized with ice‐cold methanol for 10 min. Then, cells were incubated with various primary antibodies at 4°C overnight, followed by staining with Alexa Fluor 488 goat anti‐rabbit IgG or Alexa Fluor 555 goat anti‐mouse IgG. Nucleus was stained with Hoechst 33342. Fluorescence images were acquired by Zeiss LSM 800.

### 2.10 Immunoprecipitation

The immunoprecipitation experiments were performed according to the Protein G agarose beads manual of CST company. In brief, after ice‐cold rinse with PBS, cells were lysed in 0.2 ml of lysis buffer. Equal amounts of protein (100 µg) were isolated and pre‐cleared by Protein G agarose beads (10% volume) with gentle agitation for 30 min at 4°C. The samples were incubated overnight with the antibody against NLRP3 (1:100) at 4°C. The antibody‐NLRP3 complexes were gathered with Protein G agarose beads (10% volume) with gentle rotating for 2 h at 4°C. The sediment was washed with cell lysis buffer five times, and then, the sample was resuspended by 3 × SDS loading buffer and boiled for 5 min and analysed by Western blotting.

### 2.11 MSU‐induced peritonitis and arthritis

To induce peritonitis, 10‐week‐old C57BL/6J male mice were injected intraperitoneally (i.p.) with wedelolactone (20 mg/kg, dissolved in DMSO, 10%) or vehicle 30 min before i.p. injection of MSU (1 mg MSU crystals dissolved in 0.5 ml sterile PBS). After 6 h, mice were sacrificed, and peritoneal cavities were washed with 3 ml ice‐cold PBS. Peritoneal lavage fluid was collected to assess Ly6G PerCP‐Cy5.5 and CD11b FITC antibody‐stained neutrophil by flow cytometry (BD Biosciences). IL‐1β secretion in peritoneal lavage fluid was determined by the ELISA kit.

Acute Gouty Arthritis was performed as previously described.^17^, ^37^ C57BL/6J mice were intra‐articular injections with wedelolactone (20 mg/kg), and colchicine (1 mg/kg) as positive control. One hour later, MSU (0.5 mg crystals resuspended in 20 μl sterile PBS) was administrated by intra‐articular injection. The joint swelling at the indicated time points was measured with an electronic calliper. Patella was isolated from inflamed knee joints at 24 hours after MSU injection, and cultured in Opti‐MEM containing 1% penicillin‐streptomycin for 1 h. The culture supernatant of joints was measured by Mouse IL‐1β and TNF‐α ELISA Kit.

### 2.12 H&E and immunohistochemical staining

Haematoxylin‐eosin staining (H&E) and immunohistochemical staining were performed as previously described.^36^, ^38^ Briefly, mice were sacrificed and joint tissues were isolated and fixed in 10% formalin, embedded in paraffin, sectioned at the 5 μm thickness and then stained with H&E.

For immunohistochemical (IHC) staining, the sections were deparaffinized in xylene and ethanol and rehydrated in water. Antigen retrieval was performed by heating slides with a microwave in the sodium citrate buffer for 20 minutes. Slides were quenched in hydrogen peroxide (3%) to block endogenous peroxidase activity and then washed with TBST. The primary antibodies of caspase 1 p20 and IL‐1β were incubated at 4°C overnight. The expression was detected by the SP Kit (Broad Spectrum) and DAB Horseradish Peroxidase Color Development Kit according to the manufacturer's instructions.

### 2.13 Statistical analysis

All experiments shown are representative of three independent experiments unless otherwise indicated. Values are presented as the mean ± SD. Statistical analysis was performed by the two‐tailed Student's t test for two groups or one‐way ANOVA followed by Tukey's post hoc test for multiple groups using the GraphPad Prism8 software. Data were considered statistically significant when * *P* ＜ 0.05, ** *P* ＜ 0.01, and *** *P* ＜ 0.001.

## 3 RESULTS

### 3.1 Wedelolactone suppresses the NLRP3 Inflammasome activation

**Figure 1:**
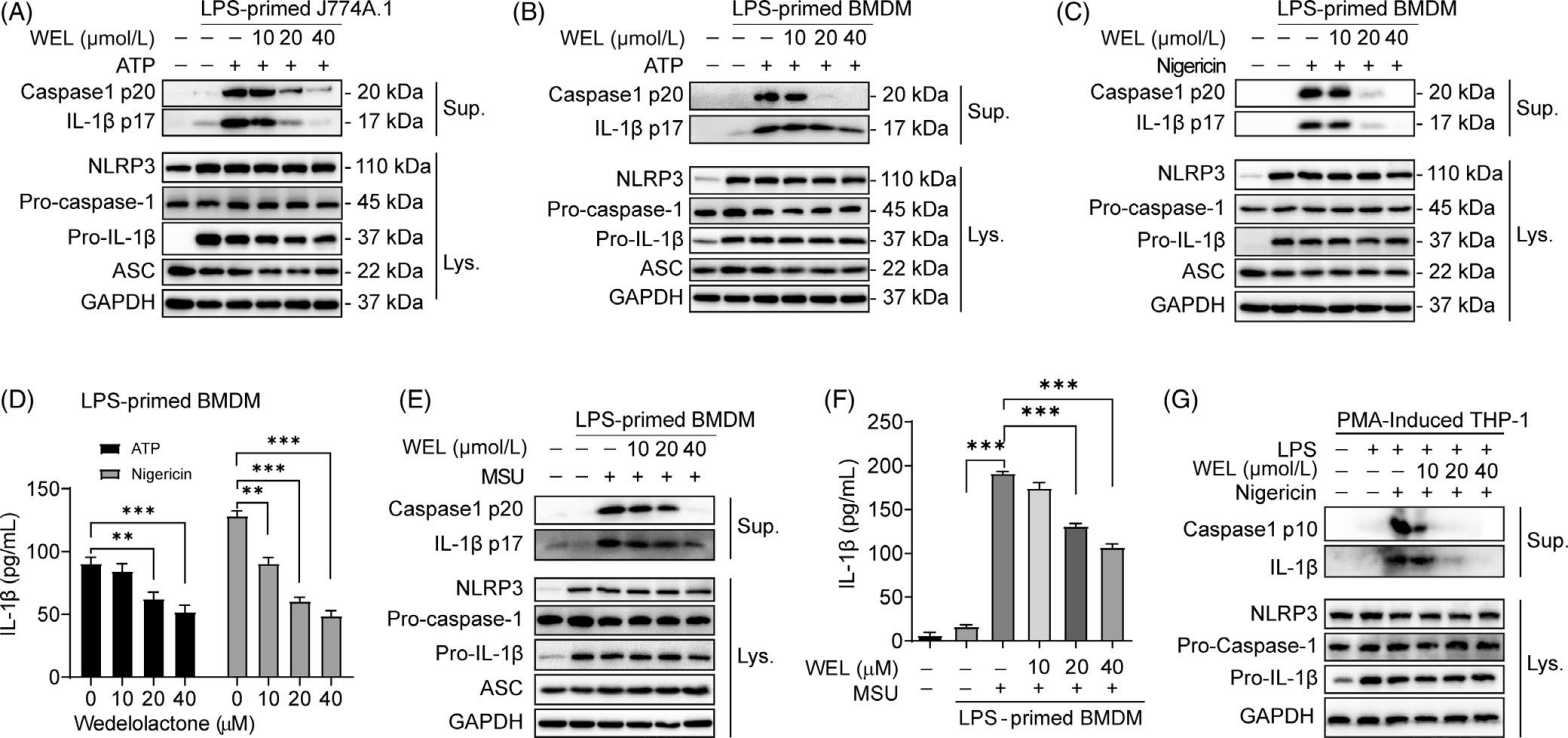
Wedelolactone inhibits NLRP3 inflammasome activation in macrophages. (A) J774A.1 was primed with LPS (500 ng/ml) for 4 h and then stimulated with ATP (3 mM) for 1 h in the presence or absence of wedelolactone. (B–F) LPS‐primed BMDMs were incubated with wedelolactone for 30 min and then stimulated with ATP (3 mM) for 1 h (B), nigericin (10 μM) for 1 h (C) or MSU (200 μg/ml) for 5 h (E). (G) PMA‐differentiated THP‐1 macrophages were primed with LPS for 4 h and then stimulated with nigericin (10 μM) for 1 h with or without wedelolactone. Supernatants (Sup.) and cell extracts (Lys.) were analysed by immunoblotting in (A‐C) and (E, G), and supernatants were also analysed by ELISA for IL‐1β release in (D) and (F). WEL, wedelolactone

To investigate the inhibitory effect of wedelolactone on NLRP3 inflammasome activation, we determine whether wedelolactone can obstruct pro‐caspase‐1 cleavage and mature IL‐1β release. Low cytotoxic dose wedelolactone (10, 20, 40 μM) was selected by Cell Counting Kit‐8 (CCK‐8) assay (Figure S1 A). The effect of wedelolactone was analysed in LPS‐primed and NLRP3 inflammasome activators (ATP, nigericin, or MSU) stimulated macrophages.^11^ Immunoblotting results showed that LPS‐primed cells express high levels of NLRP3 and pro‐IL‐1β compared with untreated group, cleaved caspase‐1 p20/p10 and mature IL‐1β (17 kDa) could be detected in the culture supernatants upon ATP, nigericin or MSU stimulation (Figure 1). Wedelolactone dose‐dependently suppressed caspase‐1 p20 and mature IL‐1β release into the supernatants of J774A.1 stimulated with ATP (Figure 1A) and BMDMs upon ATP, nigericin or MSU stimulation (Figure 1B–F). The inhibitory action of wedelolactone on active caspase‐1 p10 and mature IL‐1β secretion was further confirmed in PMA‐differentiated THP‐1 macrophages (Figure 1G).

Wedelolactone was reported to be effective in preventing NF‐κB activation.^8^, ^10^ Then, we assessed whether wedelolactone affected LPS‐induced priming for inflammasome activation by modulating NLRP3 and pro‐IL‐1β expression dependent on NF‐κB signalling. When BMDMs were treated with wedelolactone after LPS stimulation, wedelolactone did not affect LPS‐induced NLRP3 and pro‐IL‐1β expression (Figure 1A‐C, E, G). The results elucidated that the inhibiting action of wedelolactone on NLRP3 inflammasome was not modulated by NF‐κB‐mediated transcription. In contrast, while BMDMs were pre‐treated with wedelolactone for 30 min before LPS stimulation, wedelolactone suppressed LPS‐induced pro‐IL‐1β and NLRP3 expression, and blocked caspase‐1 activation as well as IL‐1β secretion (Figure S1). Thus, wedelolactone prevented both LPS priming and NLRP3 inflammasome activation in multiple macrophages. Taken together, we conclude that wedelolactone restrain NLRP3 inflammasome activation and IL‐1β release in macrophages.

### 3.2 Wedelolactone represses pyroptosis upon NLRP3 inflammasome activation

**Figure 2:**
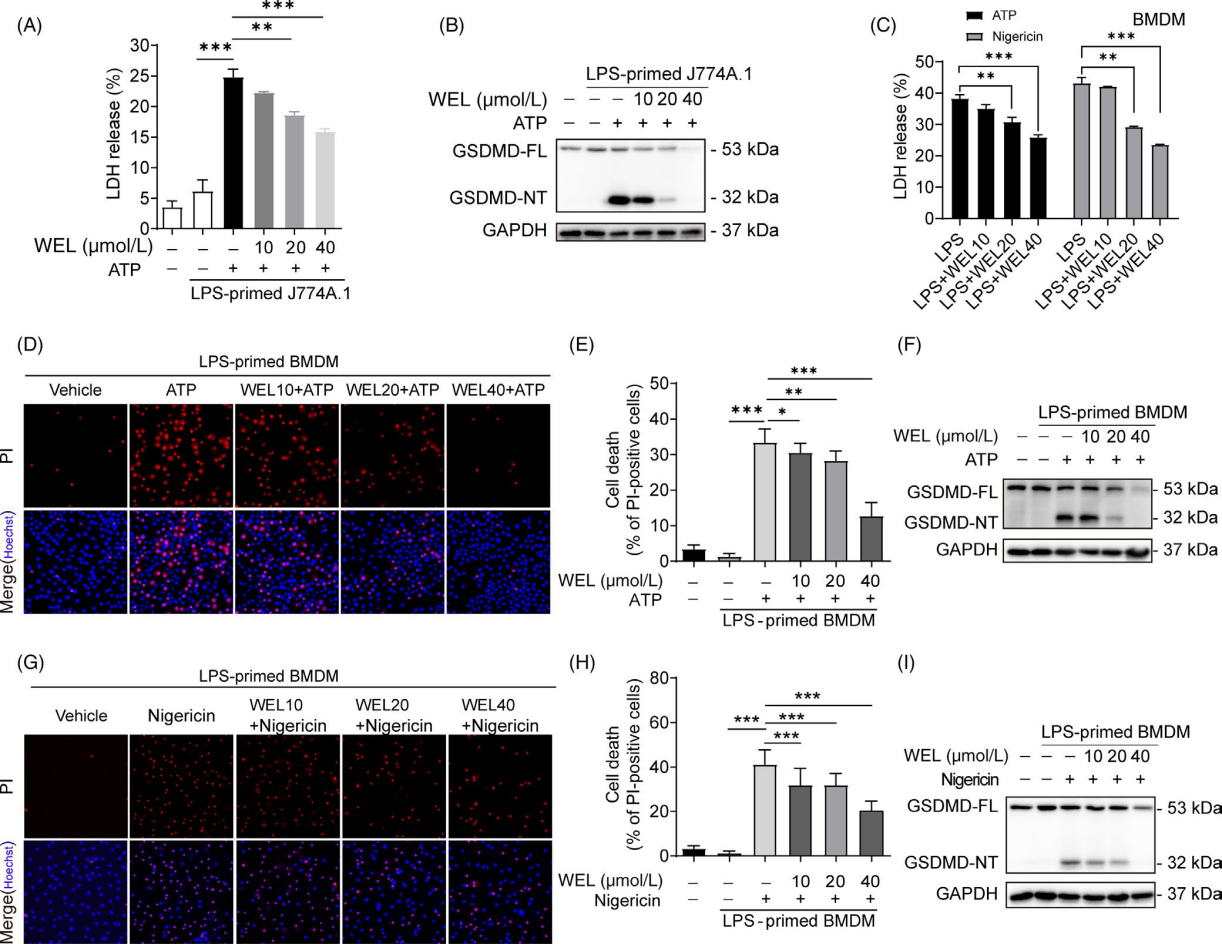
Wedelolactone restrains pyroptosis in murine macrophages. (A, B) J774A.1 primed with LPS for 4 h and stimulated with ATP for 1 h with or without wedelolactone (C–I) LPS‐primed BMDMs were incubated with wedelolactone for 30 min and then stimulated with ATP for or nigericin for 1 h. Supernatants for LDH release assay are shown in (C). (D, G) Representative immunofluorescence images of cell death were presented by PI and Hoechst 33342 staining. (E, H) The percentage of PI‐positive cells relative to all was calculated; 10 randomly chosen fields were quantified. Data are shown as mean ± SD (n = 10). (F, I) GSDMD was analysed by immunoblotting; GAPDH served as a loading control. GSDMD‐FL, full‐length GSDMD; GSDMD‐NT, GSDMD N‐terminal fragment. WEL, wedelolactone

To further investigate the inhibitory mechanisms of wedelolactone on NLRP3 inflammasome activation, BMDMs were treated with wedelolactone after LPS stimulation in the following experiments. Pyroptosis is an inflammatory cell death mediated by activation of the inflammasome. To explore the possibility that wedelolactone inhibition of IL‐1β secretion was attributed to pyroptosis, inflammatory cell death was analysed by the LDH Cytotoxicity Assay Kit. We found that wedelolactone dose‐dependently restrained ATP or nigericin‐induced LDH release in J774A.1 and BMDMs (Figure 2A, C). Inflammation mediator‐induced cell death was also stained by propidium iodide (PI). The results showed that wedelolactone dose‐dependently decreased PI‐positive cells relative to all (Figure 2D, E, G, H).

Recent studies identify that the active caspase‐1 can cleave GSDMD to generate a GSDMD‐NT fragment, which performs as an effector protein for pyroptosis.^12^, ^13^ Immunoblotting results showed that wedelolactone dose‐dependently suppressed the generation of GSDMD‐NT compared to ATP or nigericin‐activated macrophages (Figure 2B, F, I). Therefore, these results suggest that wedelolactone protects the cell from pyroptosis upon NLRP3 inflammasome activation.

### 3.3 Wedelolactone interferes with ASC assembly

**Figure 3:**
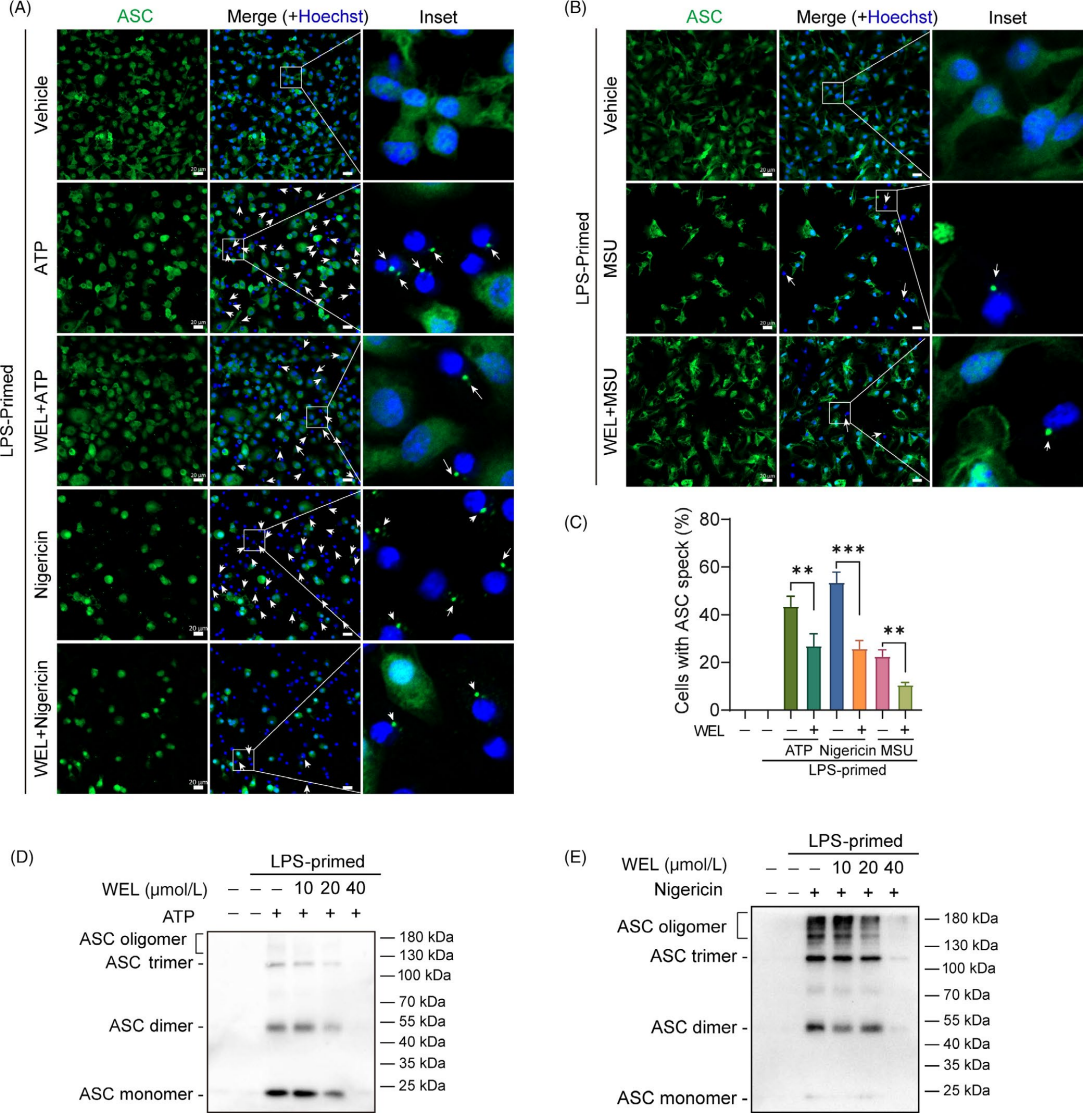
Wedelolactone blocks the assembly of ASC in macrophages. (A‐E) LPS‐primed BMDMs were incubated with wedelolactone for 0.5 h before incubated with ATP or nigericin for 1 h, or MSU for 5 h. (A, B) Representative immunofluorescence images of ASC speck formation in LPS‐primed BMDMs stimulated with ATP or nigericin or MSU in the presence or absence of wedelolactone (40 μM). White arrows indicate ASC specks (green). Scale bars, 20 μm. Quantification of macrophages containing ASC speck formation in five random images is shown in (C). (D, E) ASC oligomerization in cross‐linked cytosolic pellets was analysed by immunoblotting. WEL, wedelolactone

During the NLRP3 inflammasome activation, ASC protein oligomerizes with NLRP3 and pro‐caspase‐1 to form an active inflammasome complex. In immunofluorescence assays, ASC formed distinct specks in the cells that are hallmarks of inflammasome activation. The results showed that wedelolactone markedly reduced the formation of ASC speckles in LPS‐primed BMDMs under ATP, nigericin or MSU‐stimulating conditions (Figure 3A‐C).

Furthermore, we applied chemical cross‐linking to analyse ASC oligomerization, which also indicates the activation of NLRP3 inflammasome. Similar results were obtained by immunoblotting, wedelolactone dose‐dependently repressed ATP or nigericin‐induced ASC oligomerization in LPS‐primed BMDMs (Figure 3D, E). Taken together, these results further verify that wedelolactone disrupts NLRP3 inflammasome activation by obstructing ASC assembly to constitute ASC specks in macrophages.

### 3.4 Wedelolactone boosts NLRP3 phosphorylation on Ser/Thr residues to inhibit inflammasome activation and pyroptosis through potentiating PKA signalling

The recent studies have demonstrated that JNK and AMPK regulate the activation of NLRP3 inflammasome. But our results show that wedelolactone did not influence the activation (phosphorylation level) of JNK and AMPK (Figure S3). PKA signalling has been reported to phosphorylate NLRP3 and cause its ubiquitination degradation to restrain NLRP3 activation.^21^, ^22^ Next, we blocked the PKA’s activity using the specific inhibitor H89 to determine whether the inflammatory cell death and ASC speck formation observed in BMDMs is PKA dependent or independent.

**Figure 4:**
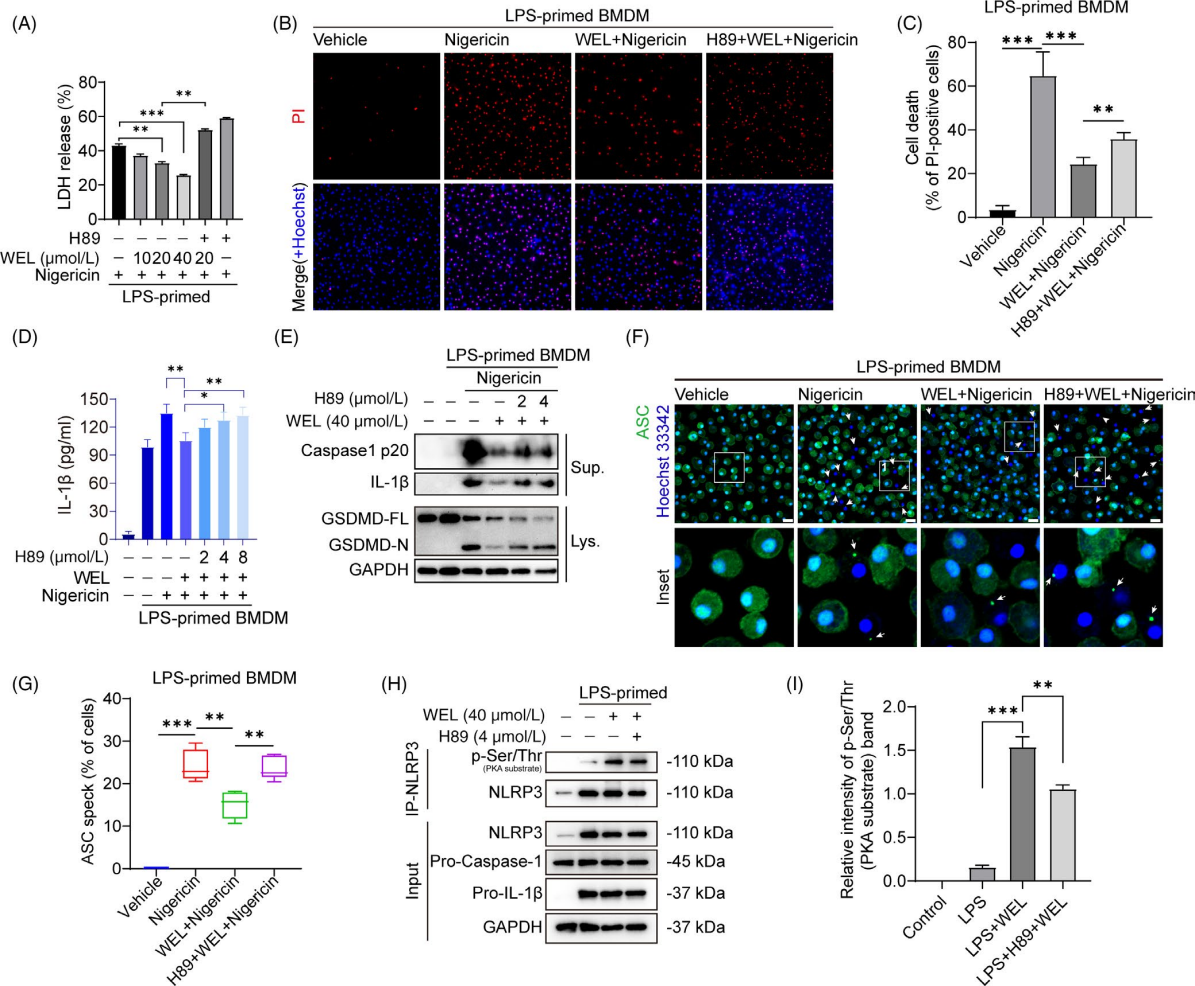
Wedelolactone promotes NLRP3 phosphorylation on Ser/Thr residues to inhibit inflammasome activation and pyroptosis depending on PKA signalling. (A‐G) LPS‐primed BMDMs were incubated with H89 for 30 min and then stimulated with nigericin with or without wedelolactone (40 μM). Supernatants for LDH release assay are shown in (A). (B) Representative immunofluorescence images of pyroptosis were indicated by PI and Hoechst 33342 staining. (C) PI‐positive cell relative to all was quantified. Data are shown as mean ± SD (n = 10 fields). ELISA of IL‐1β (D) in supernatants. (E) The supernatants concentrate and cell lysates were subjected to Western blotting with the various antibody. (F) Representative immunofluorescence images displayed ASC subcellular distribution, and the enlarged insets showed cells with an ASC speck. White arrows indicate the ASC specks (green). Scale bars, 20 µm. (G) The ASC speck formation was quantified by the cells with ASC specks relative to the total cells from five random fields each. (H and I) LPS‐primed BMDMs were pre‐treated with H89 (4 μM) for 30 min and then incubated with wedelolactone (40 μM) for 1 h. (I) The gray values of NLRP3 phosphorylation on PKA‐specific sites (p‐Ser/Thr) were quantified relative to total NLRP3 in immunoprecipitation bands (H). Sup., supernatants; Lys., cell extracts; WEL, wedelolactone

The LDH release assay results showed that H89 abrogated wedelolactone‐modulated suppression of nigericin‐induced pyroptosis (Figure 4A). The percentage of PI‐positive BMDMs reduced by wedelolactone was also upregulated by H89 (Figure 4B, C). We further tested the cleavage of caspase‐1, IL‐1β and GSDMD by immunoblotting. The blots band indicated that H89 could partly reverse the suppressive effect of wedelolactone on caspase‐1 p20, mature IL‐1β and GSDMD‐NT expression (Figure 4E). The inhibitory action of wedelolactone on nigericin‐induced ASC speck formation could also be attenuated by H89 (Figure 4F, G), further notarizing that wedelolactone modulates PKA signalling to suppress NLRP3 inflammasome activation. Immunoprecipitation results showed that the PKA‐specific phosphorylation on Ser/Thr residues of NLRP3 was greatly enhanced by wedelolactone, which can be moderately abrogated by H89 (Figure 4H, I). Collectively, we conclude that wedelolactone enhances NLRP3 phosphorylation on Ser/Thr residues to suppress inflammasome activation, pyroptosis and IL‐1β secretion through PKA signalling.

### 3.5 Wedelolactone mitigates the MSU‐induced inflammation in vivo

**Figure 5:**
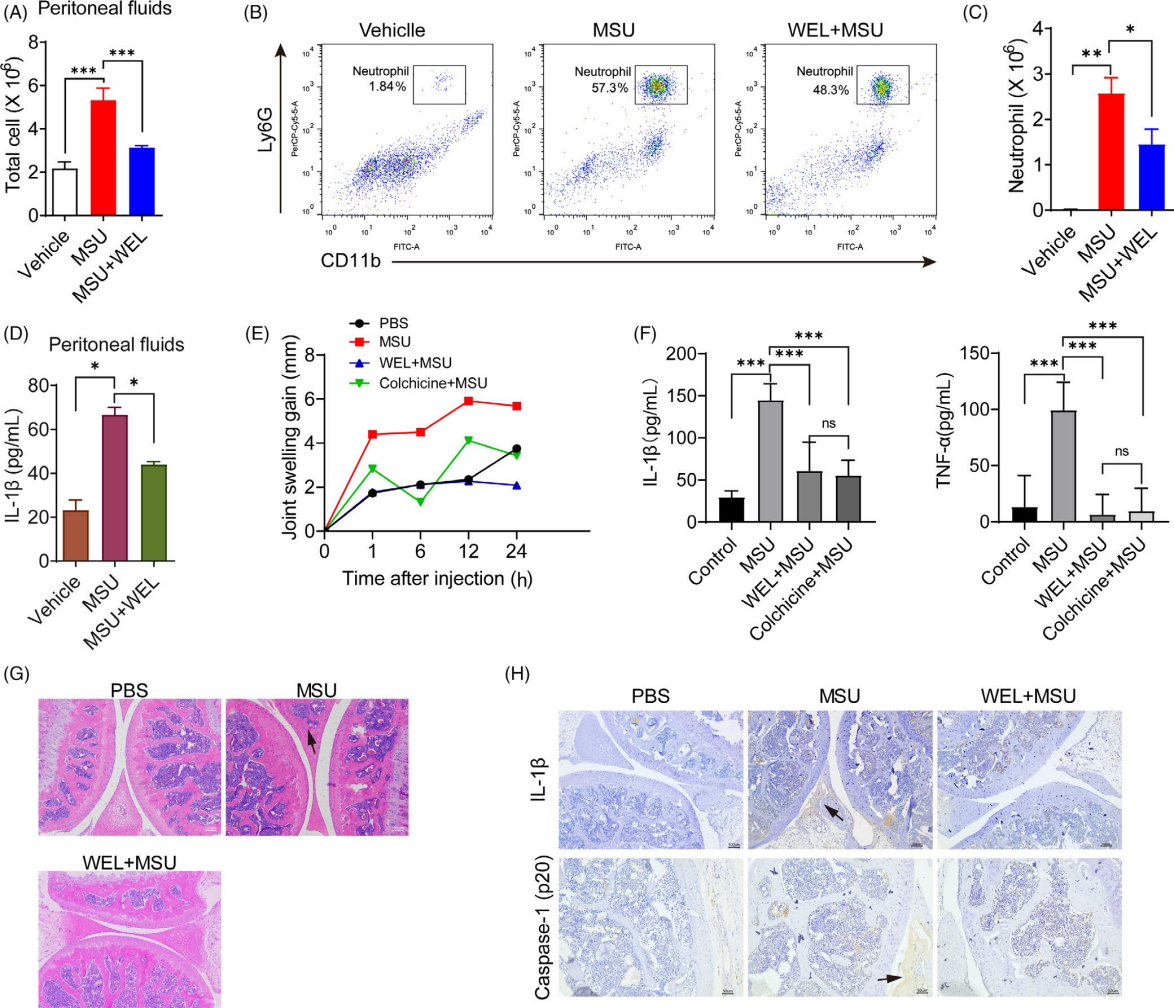
Wedelolactone suppresses MSU‐induced peritonitis and gouty arthritis. (A‐D) C57BL/6J mice were intraperitoneally injected with MSU crystals (1 mg/mouse) in the presence or absence of wedelolactone (20 mg/kg) for 6 h. (B) FACS analysis of neutrophil (CD11b + Ly6G+) numbers in the peritoneal cavity from mice; quantified data from the results are shown in (C). (D) IL‐1β in the peritoneal cavity was analysed by the ELISA kit. Data expressed as mean ± SD (n = 6 mouse). (E‐H) Mice have been intra‐articularly injected with MSU with or without wedelolactone (20 mg/kg), and colchicine (1 mg/kg) + MSU as positive control group. (E) The joint swelling gain in different time points was measured. (F) The culture supernatant of joint was measured by IL‐1β and TNF‐α ELISA kit (n = 6 mouse). (G) H&E‐stained infiltrated leucocytes (black arrow pointed) in the joints tissue. (H) IHC detection of IL‐1β and caspase 1 (p20) expression from the indicated groups. WEL, wedelolactone. Statistical differences were calculated by unpaired Student's t test: *P < .05, **P < .01, ***P < .001

As MSU crystal can activate the NLRP3 inflammasome and IL‐1β secretion to trigger the gout flare.^30^, ^39^ We next investigate whether wedelolactone could inhibit the NLRP3 inflammasome activation in MSU‐induced peritonitis and gouty arthritis in C57BL/6J mice. Intraperitoneal (i.p.) injection of MSU elicited NLRP3‐dependent inflammation characterized by IL‐1β production and massive neutrophil influx.^30^ After 6 h i.p. injection of MSU crystals, wedelolactone treatment significantly reduced MSU‐induced IL‐1β production and neutrophils (CD11b^+^Ly6G^+^) migration into the mice peritoneum compared to vehicle given group, suggesting the direct effects of wedelolactone on NLRP3‐driven peritoneal inflammation in vivo (Figure 5A‐D).

The deposition of MSU in the joints of mice leads to NLRP3 inflammasome‐dependent inflammation and pathological process.^40^ Our results showed that wedelolactone treatment alleviated MSU‐induced acute joint swelling (Figure 5E). We detected the expression of inflammatory cytokines in joints and found that wedelolactone could significantly inhibit the expression of IL‐1β and TNF‐α (Figure 5F). Although colchicine could also reduce joint swelling and the production of IL‐1β and TNF‐α, there was no significant difference between wedelolactone and colchicine (Figure 5E, F). H&E analysis demonstrated that MSU infection had caused leucocytes infiltration (black arrow pointed), but the symptoms of arthritis were alleviated by wedelolactone treatment, with fewer infiltrated leucocytes in the joints tissue sections (Figure 5G). Meanwhile, wedelolactone also significantly reduced the expression of endogenous full‐length and activated caspase‐1 (p20) and IL‐1β in the joint of MSU‐induced gouty arthritis mice (Figure 5H). Thus, we conclude that wedelolactone partially suppresses the production of MSU‐elevated IL‐1β and caspase‐1 (p20) in vivo, thereby limiting NLRP3 inflammasome activation and neutrophil influx, suggesting that wedelolactone may be a potential candidate in the treatment of gouty arthritis.

## 4 DISCUSSION

In the current study, we demonstrate that wedelolactone is a broad‐spectrum NLRP3 inflammasome inhibitor triggered by multiple stimuli. Wedelolactone enhances Ser/Thr phosphorylation of NLRP3 dependent on PKA signalling to limit inflammasome activation and pyroptosis in macrophages. Wedelolactone also exerts an inhibitory effect on NLRP3‐driven inflammation in MSU‐induced peritonitis and arthritis mice. Thus, our results unravel a previously unappreciated mechanism of wedelolactone, which might be a versatile small‐molecule tool to treat NLRP3 stimuli‐induced diseases such as gout.

We aimed to unveil the inhibitory function and molecule mechanism of wedelolactone on NLRP3 inflammasome activation and pyroptosis upon ATP, nigericin or MSU stimulation. It has been demonstrated that wedelolactone decreases the production of inflammatory cytokines such as IL‐1β through limiting NF‐kB pathway.^2^, ^10^ Consistent with the inhibitory effect of NF‐κB, the expression of NLRP3 and pro‐IL‐1β was inhibited when cells were pre‐treated with wedelolactone before LPS (Figure S1B). But wedelolactone could not notably inhibit GSDMD‐NT expression under nigericin treatment, suggesting that the inhibitory of wedelolactone on IL‐1β release might depend on the inhibition of NF‐κB mediated transcription rather than on pyroptosis (Figure S1C). Wei et al have raised that wedelolactone significantly restricted NLRP3 inflammasome activation and IL‐1β release dependent on activation of AMPK.^7^ Once NLRP3 inflammasome activated, wedelolactone facilitated ATP‐induced AMPK activation, but could not influence the AMPK signalling upon nigericin stimulation (Figure S3). The result indicates that the specific inhibition of wedelolactone on NLRP3 activation is not completely dependent on AMPK. Reactive oxygen species (ROS) can induce the activation of NLRP3 inflammasome.^11^ In support of our previous study,^41^ wedelolactone could lessen the production of mitochondrial ROS (mtROS) to exert antioxidant activity (Figure S2). We are confirmed that wedelolactone can restrict the activation of NLRP3 inflammasome and also inhibit the subsequent pyroptosis along with IL‐1β release.

Pyroptosis is mainly performed by a downstream protein of the inflammasome named GSDMD‐NT. Wedelolactone decreases cell death rate by inhibiting the generation of GSDMD‐NT, which is the active component of full‐length protein GSDMD (GSDMD‐FL) after cleavage. High concentration of wedelolactone (40 μM) could significantly inhibit both GSDMD‐FL and GSDMD‐NT than low concentration of wedelolactone (10, 20 μM) (Figure 2). This may be due to wedelolactone promoting partial degradation of GSDMD‐FL by other means, thus causing reduction of GSDMD‐NT. As wedelolactone has been shown the cytotoxicity to breast cancer cells partially though targeting proteasomal protein degradation pathway,^6^ in the future work, we will further study the effect of wedelolactone on proteasomal protein. Phosphorylation modification of inflammasome components mediates the activation or inhibition of NLRP3 inflammasome. As JNK1‐mediated NLRP3 phosphorylation facilitates NLRP3 deubiquitination to inhibit inflammasome activation.^24^ JNK modulates ASC phosphorylation and ASC speck formation.^25^ But we found that wedelolactone could not interfere the activity of JNK upon ATP or nigericin simulation (Figure S3). Recent studies have demonstrated that PKA induces Ser/Thr phosphorylation and ubiquitination degradation of NLRP3.^21^, ^22^, ^23^ We found that PKA inhibitor H89 completely reversed the inhibitory effects of wedelolactone on NLRP3 inflammasome activation and pyroptosis. Meanwhile, wedelolactone‐induced phosphorylation of NLRP3 on PKA‐specific sites was also obstructed by H89 pre‐treatment (Figure 4). Collectively, these data conclude that wedelolactone at least partly potentiates the PKA signalling to limit NLRP3 inflammasome activation and pyroptosis.

Gouty arthritis has been reported to be closely linked to NLRP3 inflammasome in joints.^29^, ^30^ Commonly used clinical anti‐gout drugs include colchicine, NSAIDS, glucocorticoids, allopurinol, benzbromarone and sodium bicarbonate. Once colchicine is long‐term used, it often brings side effects such as liver and kidney injury and gastrointestinal reactions.^42^ Importantly, we have demonstrated that wedelolactone indeed suppressed caspase 1 (p20) and IL‐1β expression in MSU‐induced gouty arthritis. As wedelolactone might inhibit both the genes expression and the inflammasome activation in vivo to reduce the production of inflammatory cytokine, we mainly focused on the anti‐inflammasome mechanism of wedelolactone, and MSU‐induced inflammation was partly conducted to verify its role in inhibiting NLRP3 activation in vivo. Whether wedelolactone could decrease serum urate levels in vivo is still unknown. Thus, more investigation is needed to facilitate our understanding of wedelolactone and improve the successful clinical application to human diseases.

In summary, our findings demonstrate that wedelolactone facilitates NLRP3 phosphorylation on Ser/Thr residues to inhibit inflammasome activation, pyroptosis and IL‐1β secretion at least partly by augmenting PKA signalling. Moreover, our results further show that wedelolactone can be used to treat MSU‐induced peritonitis and gouty arthritis probably by suppressing NLRP3 activation in vivo. Therefore, the therapeutic strategies of wedelolactone to decrease NLRP3 activity and target the pyroptosis could be useful for treating NLRP3‐driven diseases, such as gout, cancer, type 2 diabetes, Alzheimer's, Parkinson's and cardiovascular diseases.^30^, ^43^, ^44^

## CONFLICT OF INTEREST

All authors declare that they have no competing interest with respect to the contents of this article.

## AUTHOR CONTRIBUTIONS

Hao Pan, Yuqing Lin, Jianping Dou and Zhen Fu performed the most of the experiments. Yanqing Yao, Shanyu Ye and Saixia Zhang participated in the animal experiment and histological experiment. Neng Wang, Aijun Liu, Xican Li and Fengxue Zhang contributed essential reagents and manuscript revision. Hao Pan and Dongfeng Chen designed the research, analysed the data and wrote the paper.

## Supporting information

Fig S1‐S3

## Data Availability

The data that support the findings of this study are available from the corresponding author upon reasonable request.
