# Supplementary material for: Wedelolactone facilitates Ser/Thr phosphorylation of NLRP3 dependent on PKA signalling to block inflammasome activation and pyroptosis
Source: Cell Prolif. 2020 Jul 12;53(9):e12868. doi: 10.1111/cpr.12868 (PMC7507381; doi:10.1111/cpr.12868)
Supplement: Supplementary file 1 — Fig S1‐S3 [file CPR-53-e12868-s001.docx]

**Supplementary Figure**

**

**

**FIGURE S1** (A) J774A.1 cells were treated with different concentrations of wedelolactone (0.015, 0.065, 2.5, 10, 40, 160) for 24 or 48 hours, respectively. Cytotoxicity Assay was analyzed by Cell Counting Kit-8 and measured the absorbance at 450 nm using a microplate reader, and the 50% inhibition concentration (IC50) was determined from dose-response curves. (B–C) BMDMs were pre-treated with wedelolactone for 30 min followed by stimulated with LPS for 4 h and then incubated with nigericin (10 μM) for 1 h. Supernatants (Sup.) and cell extracts (Lys.) were analyzed by immunoblotting in (B). (C) IL-1β secretion were detected by ELISA Kit. WEL, wedelolactone. NIG, Nigericin.

**
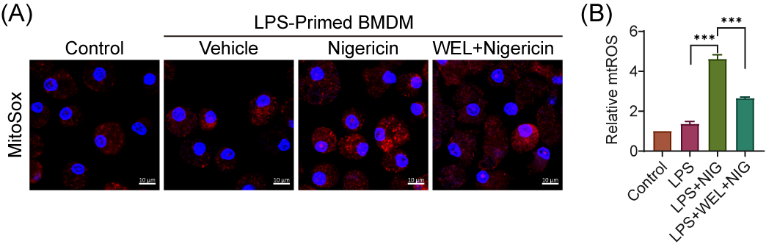
**

**FIGURE S2** (A) LPS-primed BMDMs were stimulated with nigericin (10 μM) with or without wedelolactone. Mitochondrial reactive oxygen species (mtROS) was stained with MitoSOX (red). (B) Fluorescence intensity was quantified relative to the untreated BMDMs. The values were analyzed with five random fields. Blue shows nuclei. Scale bar, 10 μm. WEL, wedelolactone. NIG, Nigericin.





**FIGURE S3** (A) LPS-primed BMDMs were treated with wedelolactone for 30 min and then incubated with ATP (3 mM) for 1 h. (B) J774A.1 primed with LPS for 4 h and stimulated with nigericin for 1 h with or without wedelolactone. Cell extracts were analyzed by immunoblotting to detect p-AMPK and p-JNK. GAPDH served as a loading control. WEL, wedelolactone.
